# Total polyunsaturated fatty acid intake and the risk of non-alcoholic fatty liver disease in Chinese Han adults: a secondary analysis based on a case–control study

**DOI:** 10.1186/s12876-021-02039-2

**Published:** 2021-11-30

**Authors:** Yong Xie, Huan Tian, Bin Xiang, Ding Li, Jian Liu, Zhuoyan Cai, Yuzhou Liu, Hua Xiang

**Affiliations:** 1 Institute of Clinical Interventional Medicine, The First Affiliated Hospital of Hunan Normal University (Hunan Provincial People’s Hospital), Changsha, 410005 China; 2 Department of Radiology, The Second Affiliated Hospital of Hebei Medical University, Shijiazhuang, 050000 China

**Keywords:** Non-alcoholic fatty liver disease, Polyunsaturated fatty acid, Case–control study, Secondary analysis, Risk

## Abstract

**Background:**

Previous studies have revealed obesity, nutrition, lifestyle, genetic and epigenetic factors may be risk factors for the occurrence and development of non-alcoholic fatty liver disease (NAFLD). However, the effect of total polyunsaturated fatty acid (PUFA) consumption on the risk of NAFLD is uncertain. Therefore, this study aimed to determine whether the total PUFA intake is independently associated with the risk of NAFLD and explore the threshold of PUFA intake better illustrate the correlation between them in Chinese Han adults.

**Methods:**

The present study was a retrospective case–control study. A total of 534 NAFLD patients and 534 controls matched by gender and age in the same center were included in this study. Using a semi-quantitative food frequency questionnaire in a health examination center in China to collect information about dietary intake and calculate nutrient consumption. A multivariate logistic regression model was used to estimate the association between total PUFA daily intake and its quartile and the incidence of NAFLD.

**Results:**

Multivariate analyses suggested a significant association between total PUFA intake and the occurrence of NAFLD. A non-linear relationship between total PUFA consumption and NAFLD risk was detected after adjusting for potential confounding factors. There was a significant connection between PUFA and the risk of NAFLD (OR: 1.32, 95% CI: 1.23–1.41, *P* < 0.0001) when PUFA intake is between 18.8 and 29.3 g/day.

**Conclusions:**

The relationship between total PUFA intake and NAFLD is non-linear. Total PUFA was positively related to the risk of NAFLD when PUFA intake is between 18.8 and 29.3 g/day among Chinese Han adults.

## Background

Non-alcoholic fatty liver disease (NAFLD), the most common cause of chronic liver disease, is a public health problem worldwide, with approximately 25% prevalence [1], and is even higher in men. According to the population studied, about 15% of adults in the general population of China suffer from NAFLD [2]. It is estimated that NAFLD will become the most common cause of liver transplantation by 2030. Previous studies have shown that obesity, nutrition, lifestyle, genetic and epigenetic factors may be closely related to the development of NAFLD [3–7]. Furthermore, it has become more and more obvious that NAFLD is a multi-system disease [8]. However, we are now facing the fact that primary care clinicians, experts, and patients still have insufficient awareness of the importance of NAFLD [9].

Fatty acids occupy an indispensable and important part of the human diet, providing the body with sufficient energy and a source of essential fatty acids (which cannot be synthesized by the body's own cells). In addition to providing the structure and regulatory functions of cell membranes, fatty acids also act as cellular messengers in signal transduction pathways, as mediators and regulators of immune function, and are also an important part of lipid transport particles (i.g., chylomicrons and lipoproteins). Fatty acids can be roughly divided into saturated fatty acids and unsaturated fatty acids. Among them, unsaturated fatty acids are roughly divided into two categories (i.e., monounsaturated fatty acids (MUFAs) and polyunsaturated fatty acids (PUFAs)). In general, PUFAs can be divided into omega-3 PUFAs (ω-3 PUFAs) and omega-6 PUFAs (ω-6 PUFAs) according to the position of the first double bond from the methyl end. It is believed that a diet rich in PUFAs are believed to be beneficial in preventing vascular accidents. Studies have shown that a diet rich in PUFAs is believed to be beneficial in preventing cardiovascular disease [10–13], bone loss in the elderly [14], Alzheimer's disease [15], common inflammatory diseases and malignant tumors (i.g., prostate cancer, colorectal cancer, breast cancer) [16–19]. However, few studies have investigated the association between PUFA and NAFLD.

In this study, we rationally used the original data of published papers to conduct secondary data analysis to determine whether total PUFA intake is independently associated with the risk of NAFLD and to explore the threshold of PUFA intake better illustrate the correlation between them.

## Methods

### Data source

We downloaded the data from the "DRYAD" database (www.Datadryad.org), which allows readers to download raw data freely. We strictly follow Dryad's terms of service, and we reasonably quoted the Dryad data set in this research. (Dryad data set: Nut intake and non-alcoholic fatty liver disease risk. Dryad Digital Repository. https://doi.org/10.5061/dryad.8nn2j46). The variables contained in the database we extracted are as follows: sex, age, total MUFA intake, total PUFA intake, nut intake, energy intake, education level, marital status, income, smoking status, body mass index (BMI), occupation, physical exercise, tea-drinking status, history of hyperlipidemia, history of diabetes, history of hypertension.

### Study population

The entire research was performed by Chinese researchers [20]. To assist us to understand their research process more clearly, we summarize the important research steps here. For specific details, we can refer to the original text. According to the report of original data, they carried out a case–control study at Health Examination Center of Affiliated Nanping First Hospital of Fujian Medical University, Nanping city, China from April 2015 to August 2017. The controls were randomly selected from the same research center and the same period. Their eligibility criteria were identical to cases (except for the requirement of liver steatosis), they were frequency-matched with cases by age (± 5 years), gender, ethnicity and region of origin. The data comes from subjects who undergo routine health examinations at the examination center. The diagnosis of NAFLD patients is based on the "Guidelines for the Diagnosis and Treatment of Non-Alcoholic Fatty Liver Disease: 2010 Update" [21]. Ultrasound examinations are implemented by experienced ultrasonographists who were unaware of laboratory and clinical data. According to the inclusion and exclusion criteria, a total of 1,068 participants were recruited and selected for data analysis. Inclusion criteria: NAFLD patients between the ages of 18–70. Exclusion criteria: (1) daily alcohol intake of > 40 g and > 20 g for men and women, respectively, (2) medical history of other liver diseases, including autoimmune hepatitis, viral hepatitis, drug-induced liver disease, etc., (3) taking blood lipid-lowering drugs or weight loss pills, (4) non-Nanping residents, (5) non-Han ethnicity, (6) extremely abnormal levels of energy intake (2 511.60 kJ [600 kcal] or 17 581.20 kJ [4200 kcal] per day for men; 2093 kJ [500 kcal]or 14,651.00 kJ [3500 kcal] per day for women). In the previously published article [20], Chen, Bingbing et al. has clearly pointed out in their original research that the research was performed in accordance with the Declaration of Helsinki, the Strengthening the Reporting of Observational Studies in Epidemiology (STROBE) Statement, and was approved by the institutional review board of Affiliated Nanping First Hospital of Fujian Medical University. The informed consent of all participants was obtained.

### Measurement of NAFLD, total PUFA intake, and other covariants

A semi-quantitative food frequency questionnaire containing 110 foods was used to collect information about participants' typical food consumption. Participants were asked to answer the average consumption frequency of the selected food: rarely, < once/month, 1–3 times/month, 1–2 times/week, 3–4 times/week, 5–6 times/week, once /day, twice/day, and > twice/day. Because each food contains the unique concentrations of PUFAs. Multiply the intake frequency of each food of the research subject by the content of PUFAs contained in the food, and then add the content of each food to obtain the total intake of PUFAs of the research subject. That is, PUFAs were calculated by multiplying the intake frequency of each food by the nutrient content of the specified portion, and summing the products of all the food items. The diagnosis of NAFLD patients is based on the above guidelines [21]. The controls matched by gender and age (± 5 years) were randomly selected from the same centre during the study period. Other covariants were described in detail in the original text.

### Statistical analysis

Continuous variables with normal distribution were presented as mean ± SD, and were compared by Student's t-test. Otherwise, continuous variables with skewed distribution were expressed as median (Q1, Q3), and were compared by the Mann–Whitney U test. Categorical variables were presented in frequency or percentage and compared using χ^2^ test or Fisher's exact test. Univariate logistic regression model was employed to assess the correlations between total PUFA intake and NAFLD risk. We performed multiple logistic regression analysis and adjusted for possible imbalances in the baseline data. Covariate screening and interaction tests were conducted, and the effects of each model were compared. Whether it is necessary to adjust the covariances according to the following principle: when added it to this model, changed the matching odds ratio by at least 0.1 [22]. In addition, we also used generalized additive models (GAM) to discern nonlinear relationships. If there was a non-linear correlation, a two-piecewise linear regression model was implemented based on the smoothing graph to calculate the threshold effect of total PUFA intake on the risk of NAFLD. When the ratio between total PUFA intake and the risk of NAFLD appears obvious in smoothed curve, the inflection point will be automatically calculated by the recursive method, and the maximum model likelihood will be employed [23]. We used the statistical R package (version 3.6.1) and EmpowerStats (http://www.empowerstats.com, X&Y Solutions, Inc., Boston, Massachusetts) to analyze all the data. Statistical significance was represented by *P* less than 0.05 (two sides).

## Results

### Baseline characteristics of the included population

**Fig. 1:**
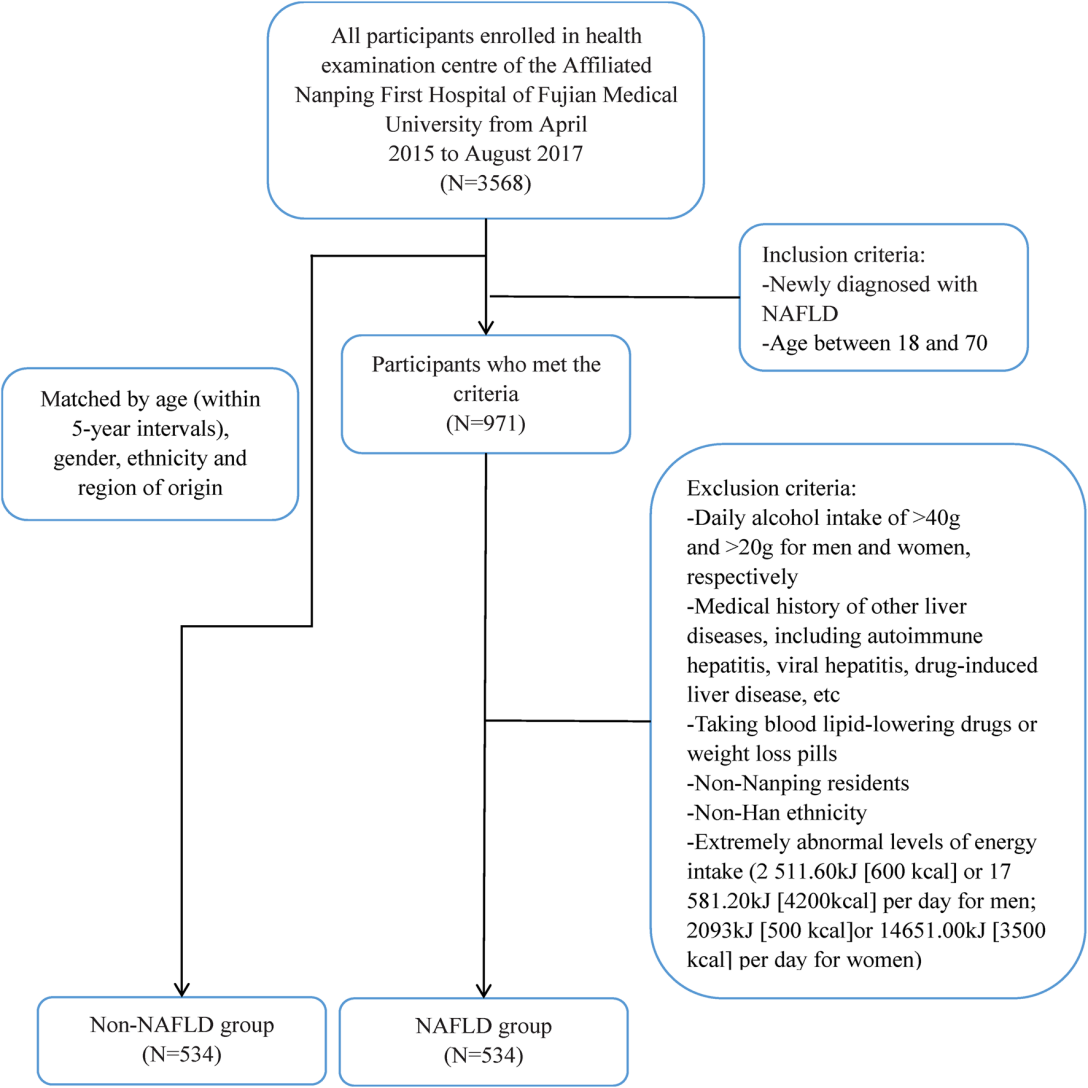
Patient selection flowchart

Of the 3568 participants from April 2015 to August 2017, 971 participants were included, and 437 were excluded from the study because they met the exclusion criteria. Finally, the case group is 534. The research flowchart is shown in Fig. 1. Table 1 showed the baseline characteristics of the population. There was no statistically significant difference in sex, age, nut intake, education level, marital status, income, smoking status, occupation, history of hyperlipidemia, history of diabetes among the different groups (Non-NAFLD vs NAFLD). Compared with the NAFLD group, patients in the Non-NAFLD group had a significantly lower MUFA intake, total PUFA intake, energy intake, overweight ratio, tea-drinking ratio, the proportion of hypertension, and a higher percentage of participants who engaged in physical exercise.

**Table 1:** Baseline Characteristics of participants (n = 1068)

Group	Non-NAFLD	NAFLD	*P*-value
Number	534	534	
MUFA intake (g/day)^‡^	31.16 (8.59)	34.12 (9.10)	< 0.001
Total PUFA intake (g/day)^‡^	23.30 (4.58)	25.93 (4.86)	< 0.001
Nut intake (g/day)^†^	2.86 (1.22–8.98)	3.15 (1.50–8.80)	0.743
Energy intake (kcal/day)^‡^	2167.63 (598.59)	2263.95 (617.49)	0.010
Sex (n, %)			1.000
Men	364 (68.16%)	364 (68.16%)	
Women	170 (31.84%)	170 (31.84%)	
Age (n, %)			0.734
< 40 year	138 (25.84%)	140 (26.22%)	
40–60	334 (62.55%)	340 (63.67%)	
≥ 60	62 (11.61%)	54 (10.11%)	
Education level (n, %)			0.332
Primary school and less than	49 (9.18%)	40 (7.49%)	
Junior middle and high school	202 (37.83%)	223 (41.76%)	
Junior college or above	283 (53.00%)	271 (50.75%)	
Marital status (n,%)			0.192
Single	62 (11.61%)	49 (9.18%)	
Married or other	472 (88.39%)	485 (90.82%)	
BMI (n, %)			< 0.001
< 18.5 kg/m^2^	20 (3.75%)	3 (0.56%)	
18.5–24.0	382 (71.54%)	179 (33.52%)	
≥ 24.0	132 (24.72%)	352 (65.92%)	
Income (n, %)			0.448
< 2000 yuan/month	35 (6.55%)	32 (5.99%)	
2000–3000	174 (32.58%)	157 (29.40%)	
≥ 3000	325 (60.86%)	345 (64.61%)	
Smoking status (n, %)			0.284
Never smoker	383 (71.72%)	367 (68.73%)	
Smoker	151 (28.28%)	167 (31.27%)	
Tea-drinking status (n, %)			0.029
No drinking	234 (43.82%)	199 (37.27%)	
Drinking	300 (56.18%)	335 (62.73%)	
Occupation (n, %)			0.187
Mental labour	152 (28.46%)	158 (29.59%)	
Physical labour	136 (25.47%)	111 (20.79%)	
Other	246 (46.07%)	265 (49.63%)	
Physical exercise (n, %)			0.046
Light	156 (29.21%)	194 (36.33%)	
Moderate	164 (30.71%)	147 (27.53%)	
Severe	214 (40.07%)	193 (36.14%)	
History of hyperlipidemia (n, %)			0.661
No	508 (95.13%)	511 (95.69%)	
Yes	26 (4.87%)	23 (4.31%)	
History of diabetes (n, %)			0.241
No	519 (97.19%)	512 (95.88%)	
Yes	15 (2.81%)	22 (4.12%)	
History of hypertension (n, %)			< 0.001
No	436 (81.65%)	375 (70.22%)	
Yes	98 (18.35%)	159 (29.78%)	

*NAFLD* non-alcoholic fatty liver, *BMI* body mass index, *MUFA* monounsaturated fatty acid, *PUFA* polyunsaturated fatty acid

^†^Medians (IQRs)

^‡^Mean (SD)

### Univariate and multivariate analysis

We used univariate logistic regression model to evaluate the correlations between total PUFA intake and the risk of NAFLD (Table 2). At the same time, we showed the non-adjusted and adjusted models in Table 3. In the crude model, total PUFA intake showed positive correlation with NAFLD risk (odds ratio (OR): 1.13, 95% confidence interval (CI): 1.10–1.17), *P* < 0.0001). In the minimally adjusted model (adjusted sex, age), the effect size did not have obvious changes (OR: 1.14, 95% CI: 1.11–1.18, *P* < 0.0001). In addition, the result still did not have obvious changes (OR: 1.18, 95% CI: 1.13–1.23, *P* < 0.0001) in the fully adjusted model. Then, we also conducted a sensitivity analysis. After converting total PUFA intake as a categorical variable (Quartile), we also observed the same significant positive trend (*P* for trend was < 0.0001).

**Table 2:** The results of univariate analysis

	Statistics	OR (95% CI)	*P-*value
Sex			
Men	728 (68.16%)	Ref	
Women	340 (31.84%)	1.00 (0.77, 1.29)	1.000
Age			
< 40 year	278 (26.03%)	Ref	
40–60	674 (63.11%)	1.00 (0.76, 1.33)	0.9809
≥ 60	116 (10.86%)	0.86 (0.56, 1.33)	0.4909
Education level			
Primary school and less than	89 (8.33%)	Ref	
Junior middle and high school	425 (39.79%)	1.35 (0.85, 2.14)	0.1974
Junior college or above	554 (51.87%)	1.17 (0.75, 1.84)	0.4866
Marital status			
Single	111 (10.39%)	Ref	
Married or other	957 (89.61%)	1.30 (0.88, 1.93)	0.1933
BMI			
< 18.5 kg/m^2^	23 (2.15%)	Ref	
18.5–24.0	561 (52.53%)	3.12 (0.92, 10.65)	0.0687
≥ 24.0	484 (45.32%)	17.78 (5.20, 60.82)	< 0.0001
Income			
< 2000 yuan/month	67 (6.27%)	Ref	
2000–3000	331 (30.99%)	0.99 (0.58, 1.67)	0.9608
≥ 3000	670 (62.73%)	1.16 (0.70, 1.92)	0.5605
Smoking status			
Never smoker	750 (70.22%)	Ref	
Smoker	318 (29.78%)	1.15 (0.89, 1.50)	0.2845
Tea-drinking status			
No drinking	433 (40.54%)	Ref	
Drinking	635 (59.46%)	1.31 (1.03, 1.68)	0.0293
Occupation			
Mental labour	310 (29.03%)	Ref	
Physical labour	247 (23.13%)	0.79 (0.56, 1.10)	0.1575
Other	511 (47.85%)	1.04 (0.78, 1.37)	0.8043
Physical exercise			
Light	350 (32.77%)	Ref	
Moderate	311 (29.12%)	0.72 (0.53, 0.98)	0.0363
Severe	407 (38.11%)	0.73 (0.54, 0.97)	0.0281
History of hyperlipidemia			
No	1019 (95.41%)	Ref	
Yes	49 (4.59%)	0.88 (0.50, 1.56)	0.6610
History of diabetes			
No	1031 (96.54%)	Ref	
Yes	37 (3.46%)	1.49 (0.76, 2.90)	0.2442
History of hypertension			
No	811 (75.94%)	Ref	
Yes	257 (24.06%)	1.89 (1.42, 2.51)	< 0.0001
MUFA intake	32.64 ± 8.97	1.04 (1.02, 1.05)	< 0.0001
Total PUFA intake	24.62 ± 4.90	1.13 (1.10, 1.17)	< 0.0001
Nut intake	8.01 ± 12.97	1.00 (0.99, 1.01)	0.7431
Energy intake	2215.79 ± 609.74	1.00 (1.00, 1.00)	0.01

*BMI* body mass index, *MUFA* monounsaturated fatty acid, *PUFA* polyunsaturated fatty acid, *OR* odds ratio, *Ref* reference

**Table 3:** Relationship between total PUFA intake and the risk of NAFLD in different models

Variable	Non-adjusted (OR, 95% CI, *P*)	Adjust I (OR, 95% CI, *P*)	Adjust II (OR, 95% CI, *P*)
Total PUFA intake (g/day)	1.13 (1.10, 1.17) < 0.0001	1.14 (1.11, 1.18) < 0.0001	1.18 (1.13, 1.23) < 0.0001
Total PUFA intake (g/day) (Quartile)			
Q1	Ref	Ref	Ref
Q2	2.04 (1.42, 2.92) 0.0001	2.21 (1.52, 3.20) < 0.0001	2.57 (1.69, 3.90) < 0.0001
Q3	3.09 (2.17, 4.41) < 0.0001	3.45 (2.39, 4.98) < 0.0001	3.84 (2.49, 5.93) < 0.0001
Q4	5.28 (3.65, 7.64) < 0.0001	6.07 (4.12, 8.93) < 0.0001	8.30 (4.87, 14.13) < 0.0001
*P* for trend	< 0.0001	< 0.0001	< 0.0001

Non-adjusted model adjust for: None

Adjust I model adjust for: age, sex

Adjust II model adjust for: age, sex, nut intake, energy intake, BMI, tea-drinking status, history of hypertension, MUFA intake, physical exercise, education, marital status

Abbreviations as in Tables 1 and 2

### The results of the two-piecewise linear regression model

**Fig. 2:**
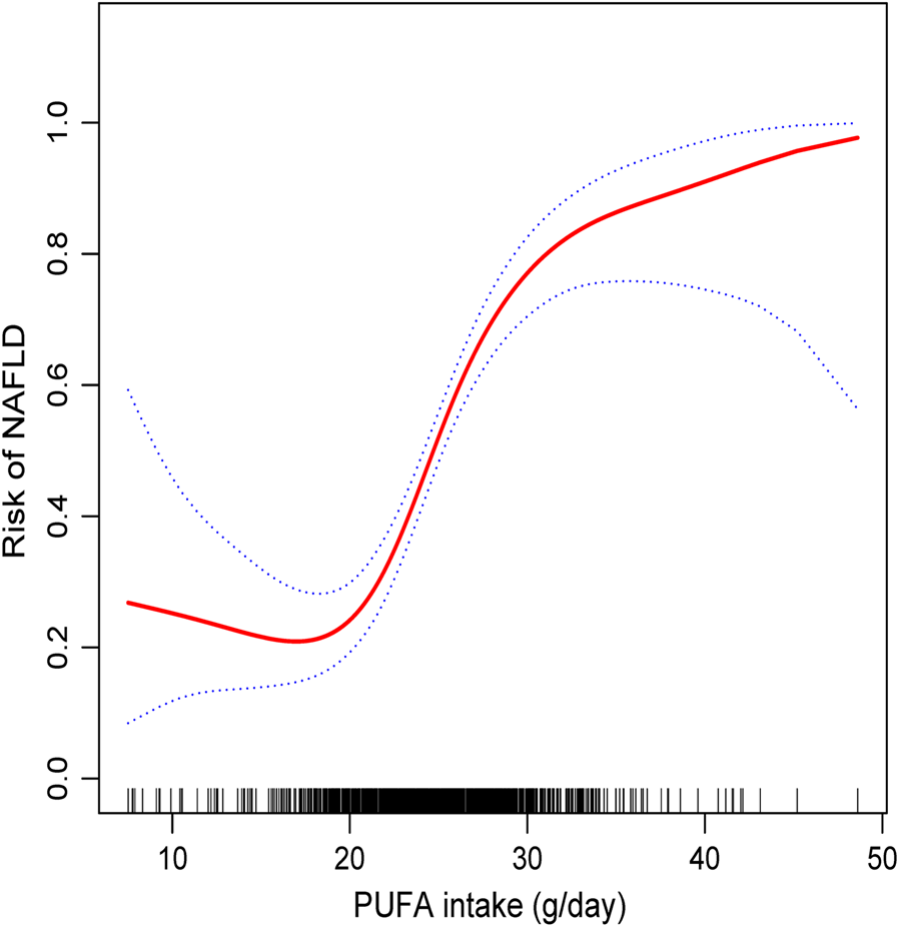
The relationship between total PUFA intake and the risk of NAFLD. A nonlinear relationship between them was detected after adjusting for age, sex, nut intake, energy intake, BMI, tea-drinking status, history of hypertension, MUFA intake, physical exercise, education, marital status. The total PUFA intake is not connected with the risk of NAFLD when inflection point is < 18.8 g/day or > 29.3 g/day. Conversely, the total PUFA intake ranged between 18.8 and 29.3 g/day and showed a significant correlation with the risk of NAFLD. The risk of NAFLD increases as the total PUFA intake increases

Since total PUFA intake was a continuous variable, it is inevitable to analyze the nonlinear relationship. As shown in Fig. 2 in our study, the relationship between total PUFA intake and NAFLD risk was non-linear, which is obvious (after adjusting sex, age, nut intake, energy intake, BMI, tea-drinking status, hypertension, MUFA intake, physical exercise, education, marital status). By using a two-piecewise linear regression model, we calculated the inflection point were 18.8 and 29.3, respectively. On the left of 18.8 and on the right of 29.3 inflection point, the effect size, 95% CI and *P* value were 0.91, 0.81–1.02 and 0.1045; 1.13, 0.99–1.30 and 0.0756, respectively. Total PUFA was positively related to the risk of NAFLD when PUFA intake is between 18.8 and 29.3 g/day. With each additional unit of PUFA, there was a 0.32 times increase in the risk of NAFLD (OR: 1.32, 95%CI: 1.23–1.41, *P* < 0.0001) (Table 4).

**Table 4:** The results of two-piecewise linear regression model

Inflection point of total PUFA intake (g/day)	Effect size (OR)	95%CI	*P-*value
< 18.8	0.91	0.81 to 1.02	0.1045
18.8–29.3	1.32	1.23 to 1.41	< 0.0001
> 29.3	1.13	0.99 to 1.30	0.0756

Effect: NAFLD risk, Cause: Total PUFA intake

Adjusted: age, sex, nut intake, energy intake, BMI, tea-drinking status, history of hypertension, MUFA intake, physical exercise, education, marital status

Abbreviations as in Table 2

The association between total PUFA intake to energy intake ratio and NAFLD risk.

The association between total PUFA intake to energy intake ratio (henceforth PUFA/Energy) and NAFLD risk was also investigated. As shown in Table 5, the risk of NAFLD increased as PUFA/Energy increased (OR: 1.14, 95% CI: 1.08–1.20, *P* < 0.0001).

**Table 5:** Relationship between total PUFA intake to energy intake ratio and the risk of NAFLD in different models

Variable	Non-adjusted (OR, 95%CI, *P*)	Adjust I (OR, 95%CI, *P*)	Adjust II (OR, 95%CI, *P*)
Total PUFA intake to energy intake ratio (g/1000 kcal)	1.08 (1.04, 1.12) 0.0001	1.10 (1.15, 1.15) < 0.0001	1.14 (1.08, 1.20) < 0.0001

Non-adjusted model adjust for: None

Adjust I model adjust for: age, sex

Adjust II model adjust for: age, sex, nut intake, BMI, tea-drinking status, history of hypertension, MUFA intake, physical exercise, education, marital status

Abbreviations as in Table 2

## Discussion

This study aimed to explore the relationship between total PUFA intake and NAFLD among participants. To our knowledge, this is the first research reported that the relationship between PUFA consumption and the risk of NAFLD and explored the threshold of PUFA daily intake. As is shown in fully adjusted model, total PUFA intake is positively correlated with NAFLD risk even analyzed by sensitivity analysis. We also found on the left of 18.8 inflection point and the right of 29.3 inflection point, the effect size, 95%CI and *P* value were 0.91, 0.81–1.02 and 0.1045; 1.13, 0.99–1.30 and 0.0756, respectively. However, we observed a significant positive association between total PUFA intake and NAFLD risk when the inflection point is between 18.8 and 29.3 (Fig. 2, Table 4).

We simultaneously used the following keywords—“polyunsaturated fatty acid” and “non-alcoholic fatty liver disease” to conduct a comprehensive search on the PubMed database. Forty-eight scientific publications were retrieved on database as of the end of May 26, 2021, but only 12 of them can be used as references for our research. Previous studies found consuming ω-3 PUFAs may improve liver steatosis and reduce liver enzyme parameters [24] and reduce liver fibrosis [25], which is consistent with a recent paper conducted by Lee CH et al. [26], furthermore, the latter also indicated that ω-3 PUFAs consumption may also improve blood lipid levels and obesity. In one study performed by Parker HM et al. [27], they showed ω-3 PUFA intake can ameliorate liver fat and liver function, which is consistent with the research conclusion of Guo et al. [28]. Further subgroup analysis found compared with non-randomized controlled trials (non-RCTs), RCTs showed PUFA has a greatly significant benefit in improving liver fat [27]. However, the authors of the article pointed out that the appropriate dosage of ω-3 PUFA intake is currently not known. Besides, one latest RCT [29] found ω-3 PUFA had no beneficial effects on liver enzymes, lipid profile, insulin resistance. Also, another RCT with a sample size of 50 conducted by Parker HM et al. [30] indicated ω-3 PUFAs do not seem to be an effective drug for reducing liver fat in overweight men. This indicates that bulkier, well-designed RCTs are urgently necessary to verify the impact of ω-3 PUFA on the above parameters and we need to consume ω-3 PUFAs according to the individual level to promote body health. In our current study, we have observed as the total intake of PUFA per unit increases, the risk of NAFLD increases when the inflection point range is between 18.8 and 29.3 (Table 4), which suggests when the total daily intake of PUFA exceeds 18.8 g, the risk of NAFLD comes with it. However, the original study did not separately calculate the intake of omega-3 or omega-6 PUFAs, instead calculated the overall PUFA intake. Also, the ratio of ω-3/ω-6 is important but not mentioned in the present study. Instead, we just explored the total PUFA intake, and did not solve the problem of what the appropriate ratio is. Of course, in the future, a larger sample and more rationally designed research are necessary to confirm our conclusions and discuss the most appropriate ratio. Here, we have only conducted a preliminary exploration of this.

Considering that PUFAs are included in various foods, it is reasonable to think that PUFA intake is positively correlated with overall food consumption. Therefore, we suspected that the association between PUFA intake and NAFLD risk may just simply be due to this positive correlation. More thorough studies would be required to negate this possibility, so we carried out further verification (i.e., dividing PUFAs by energy to get the ratio, and analyzed the relationship with NAFLD risk). Finally, we discovered the risk for NAFLD increased as PUFA/Energy increased (OR:1.14, 95% CI: 1.08–1.20, *P* < 0.0001).

Although PUFAs are essential nutrients for the human body and the appropriate dosage is beneficial to the human multiple systems, the maximum daily intake dose is lacking research confirmation. Furthermore, since the original publication did not record the intake of omega-3 or omega-6 PUFA and the ratio of ω-3/ω-6 [20]. So our study did not analyze them separately but calculated the total PUFA intake. Taking into account our research conclusions, we cautiously recommend that the daily intake of PUFAs should be around 18.8 g, which should not be higher than this critical value. As for the appropriate proportion of omega-3 or omega-6 PUFA in 18.8 g, we temporarily lack evidence to support it.

Our research has several advantages. Firstly, this is the first study to explore the association between the PUFA and NAFLD and explore the threshold of total PUFA daily intake. PUFAs are essential nutrients for the human body. The appropriate dosage is beneficial to the human cardiovascular system, nervous system, etc., but the maximum daily intake dose is lacking research confirmation, therefore, the findings of this study provide a new reference for the primary prevention of NAFLD. Secondly, we employ a GAM to fit the nonlinear relationship between total PUFA intake and NAFLD risk. GAM has unique advantages in dealing with non-linear relationships, and can handle non-parametric smoothing and fitting regression splines to data. Making good use of GAM will help us better discover the true relationship between exposure and results. Thirdly, this research is an analytical case–control study with large sample size and will include inevitable potential confounding factors. However, we used strict statistical adjustments to minimize potential or residual confounding. Finally, the positive correlation between PUFA and the risk of NAFLD is stable, so the conclusions of this study can be considered relatively reliable. Of course, our research also has some limitations. Firstly, due to the nature of case–control study, potential bias may exist. Secondly, this paper is the first to uncover the relationship between PUFA and the risk of NAFLD, so there is a lack of comparison with similar studies and mutual verification of related basic research. Therefore, the conclusions of this study should be carefully considered, and a larger sample of RCTs should be conducted in the future to verify this conclusion. Thirdly, because the study population comes from Chinese Han adults (i.e., Nanping residents of China), it may not be applicable to individuals of other areas or races, but it can be used as a reference. Fourthly, since we are a secondary analysis of the data of Chen, Bingbing et al. [20], the diagnosis of NAFLD was confirmed only by abdominal ultrasound examination, not a biopsy. However, the biopsy is the gold standard method for diagnosing NAFLD staging. Abdominal ultrasound has low sensitivity in detecting mild steatosis [31], which means NAFLD populations are highly likely to be classified as non-NAFLD ones. Furthermore, some risk factors such as cholecystectomy [32], thyroid-stimulating hormone level [33] cannot be included in the analysis. Last but not least, since the original publication did not record the intake of omega-3 or omega-6 PUFA, so our study did not analyze them separately. Besides, the ratio of ω-3/ω-6 is important, but it was not mentioned in this study. On the contrary, we only discussed the total PUFA intake, and did not solve the problem of what proportion is appropriate.

## Conclusion

The relationship between total PUFA intake and NAFLD is non-linear. Total PUFA was positively related to the risk of NAFLD when PUFA intake is between 18.8 and 29.3 g/day among Chinese Han adults.

## Data Availability

Data can be downloaded from the ‘DATADRYAD’ database (https://doi.org/10.5061/dryad.8nn2j46).

## Abbreviations

NAFLD: Non-alcoholic fatty liver disease
MUFAs: Monounsaturated fatty acids
PUFAs: Polyunsaturated fatty acids
BMI: Body mass index
GAM: Generalized additive models
OR: Odds ratio
CI: Confidence interval
non-RCTs: Non-randomized controlled trials
